# Polymer architecture effect on rheology and segmental dynamics in poly (methyl methacrylate)-silica nanocomposite melts

**DOI:** 10.55730/1300-0527.3576

**Published:** 2023-06-23

**Authors:** Saeid DARVISHI, Erkan ŞENSES

**Affiliations:** 1 Department of Chemical and Biological Engineering, Koç University, İstanbul, Turkiye; 2 Koç University Boron and Advanced Materials Application and Research Center (KUBAM), İstanbul, Turkiye; 3 n2STAR-Koç University Nanofabrication and Nanocharacterization Center for Scientific and Technological Advanced Research, İstanbul, Turkiye

**Keywords:** Polymer nanocomposites, chain architecture, polymer topology, NP dispersion, rheology

## Abstract

Architecturally different polymer chains lead to fundamentally different rheological responses and internal dynamics, which can be utilized to rationalize advanced thermoplastic nanocomposites with tunable mechanical behavior. In this work, three model poly (methyl methacrylate) (PMMA) polymers with linear, bottlebrush, and star architectures with the same total molar mass were investigated in their neat form, and nanocomposites with well-dispersed silica nanoparticles using rheology and broadband dielectric spectroscopy (BDS). The master curves of the dynamic moduli obtained by time-temperature superposition (TTS) over the entire range from the Rouse regime to the terminal flow and a sequence of significantly different relaxation modes were observed for the samples with linear and branch chains. While linear chains form an entangled polymer network, the branched bottlebrush, and star chains show a viscoelastic response with no sign of rubbery entanglement plateau and a weak arm relaxation regime between Rouse and terminal flow, akin to other branched polymers. Moreover, branched chains showed a higher fragility index (m = 3.46 for the bottlebrush and 5.36 for the star) compared to linear chains (m = 3.29) due to dynamical heterogeneities induced by arm relaxation. The addition of nanoparticles affects only the terminal relaxation regime, where the whole chain motion is hindered by the attractive nanoparticles. The dynamics of the polymer segment were investigated by performing broadband dielectric spectroscopy (BDS) at a frequency range from 10^−2^ Hz to 10^7^ Hz. The results revealed more than 10 times slower segmental relaxation for the star homopolymers and a slowdown in the α-relaxation process for all three architectures in their composite form. The dynamical slowdown in the composites is temperature dependent and more pronounced at low temperatures (leading to approximately equal to 80 times slower dynamics for nanocomposite with bottlebrush PMMA at 150 °C) due to prolonged relaxation of the interfacial polymer compared to the matrix chains. The results from this study have practical applications in fields such as gas separation and polymeric electrolyte membranes, where simultaneous improvement of segmental mobility and mechanical moduli is highly desired.

## 1. Introduction

Polymer nanocomposites (PNCs) with tunable and superior properties are crucial for the development of advanced technologies [1]. Polymer chains and nanoparticles interact at varying strength and length scales and control the thermo-mechanical properties of PNCs [2–4]. Regarding the nanoparticles (NPs), their chemistry, size, concentration, shape, and state of dispersion play a critical role in the final properties of polymer-nanoparticle composite systems [4–7]. On the polymer side, molecular weight, chain rigidity, segment packing density, grafting density, and chemical and dynamical heterogeneity are known to influence the local and bulk rheological properties of PNCs [2, 4, 8–10]. The role of chain architecture, which controls the internal dynamics of the polymers and their interaction with NPs, is not well-known.

Investigating the effects of polymer architecture in PNCs requires achieving uniform dispersion of NPs in the polymer matrices. This is highly affected by production methods. Indeed, nanocomposite preparation techniques play a critical role in the development of PNCs with desirable properties [11–13]. When the NP dispersion is poor, the resulting composite material may exhibit reduced mechanical strength, poor electrical conductivity, or other undesirable properties. To address this challenge, a variety of sample preparation techniques have been developed to achieve a high degree of NP dispersion within polymer matrices. Melt-mixing, solution-mixing, in situ polymerization, freeze drying, electrospinning, and selective laser sintering techniques are the most commonly used techniques to produce polymer nanocomposites with a variety of NP materials, including silica particles, metal nanoparticles, and carbon nanotubes [12]. For the PMMA-silica system, the solvent casting method is known to provide good NP dispersion [14], this method is being used in this study to prepare the samples.

The majority of the PNC studies utilize linear architecture (mainly due to difficulties in the synthesis of complex architectures) where the molecular weight, M_w_, of the polymer determines the overall properties [15, 16]. In nonlinear polymers with branch structures, however, M_w_ is not the only controlling main factor. For example, polymers with star-shaped architecture have several cocentric branches where the length and number of branches determine the structure of the chain which can be tuned from the hard sphere for stars with a high number of arms (functionality) and short chain length, to soft coils for stars with low functionality and/or long arms [17, 18]. Bottlebrush polymers are macromolecules with polymeric side chains on each repeat unit and their conformation and physical properties are controlled by their grafting density and side chain length [19]. This specific structure causes steric crowding among closely packed branches and reduces the flexibility of the backbone. Therefore, only bottlebrush macromolecules with high molar mass (orders of magnitudes higher than entanglement molar mass for linear chains) and long backbone chain length can entangle in the melt [20, 21]. The unique structures of branched polymers lead to a number of novel and potentially useful properties that allow them to find potential applications in a variety of fields, such as tissue engineering, drug delivery, and polymer electrolytes [13, 22–29].

The linear viscoelastic properties of branched homopolymer polymer melts, such as stars [17, 30, 31], combs [32, 33], H-polymers [34], and bottlebrush [20, 21, 35], have been studied in detail in the past few decades. The dynamics of these systems are often described by a hierarchical relaxation process in which the side chains relax at the intermediate time scale after the relaxation of polymer segments in the glassy regime and the entire polymer chain relaxes at longer times [20]. The plateau of the side chain relaxation depends on the length of the branches, and for branched polymers with very short side chains, this mode is not observed in their dynamic master curves [36]. Even though the nonlinear, NP-free, homopolymers are studied in detail in the literature, a comprehensive understanding of the effect of chain architecture on the mechanical properties of nanoparticle-filled polymers is lacking.

A previous study of the authors showed in a PEO/silica composites system that changing polymer chain architecture from linear to hyperbranched and star without altering the total molecular weight, NP concentration, and state of dispersion, slows down the average segmental dynamics of polymer chains, resulting in different levels of mechanical reinforcement in the terminal flow regime, and accelerated nanoscale motion of NPs in the matrices of branched polymers [4]. However, PEO is a low *T*_g_ polymer (*T*_g_ approximately −47 °C), with a semicrystalline structure with melting temperature *T*_m_ approximately 65 °C, which makes it difficult to investigate the faster dynamics such as reptation, chain diffusion, and cage relaxations in its melt state. Therefore, it was not possible to monitor the changes in these intermediate-level dynamics using the PEO-Silica system with rheometry or other spectroscopic techniques. In this work, high *T*_g_ PMMA polymers (*T*_g, PMMA_ approximately 120 °C) with linear, bottlebrush, and star architectures in their neat and composite forms were employed to probe the effect of polymer chain architecture on the entire relaxation spectra. Small-angle X-ray scattering (SAXS) and scanning electron microscope (SEM) imaging were used to investigate the structure of the NP network. Differential scanning calorimetry (DSC) was used to obtain glass-transition temperatures. The dynamics of the nanocomposites and polymers were probed using small amplitude oscillatory shear (SAOS) and broadband dielectric spectroscopy (BDS). The results show fundamental differences in the rheological behavior of neat nonlinear homopolymers and their PNCs in the melt state from those made from linear chains with the same total molar mass, same NP concentration, and dispersion state. Our results suggest that polymer chain architecture can be used as a decisive tuning parameter to alter the mechanical properties of polymer nanocomposites.

## 2. Materials and methods

### 2.1. Materials

PMMA with linear, 16-arm star, and bottlebrush architectures and total molecular weights of approximately equal to 120 kDa were used. Linear PMMA was purchased from Sigma-Aldrich and used as received. Star and bottlebrush (BB) PMMA were synthesized in the Center for Nanophase Materials at Oak Ridge National Laboratory (ORNL). Star polymers have 16 arms each having approximately equal to 7 kg/mol, whereas the bottlebrush has 15 side chains (with a degree of polymerization of about 50) connected along a polynorbornene backbone. For all polymers, the dispersity is lower than 1.4 as provided by the manufacturers. Colloidal silica NPs with an average diameter of 50 nm and dispersed in methyl ethyl ketone (MEK) were supplied by Nissan Chemical America and used as received (NP sizes reported here are measured by DLS). Acetonitrile (CH_3_CN) with a grade >99.9% was purchased from IsoLab and used to dissolve the polymers and prepare the PNCs.

### 2.2. Sample preparation

Similar to the method used by Ref. [14], first, the PMMA polymers were dissolved in acetonitrile (CH_3_CN) in glass vials at 150 mg/mL concentration. Then, the polymer solutions were stirred for 12 h with a magnetic stir bar and bath sonicated for 15 min. The proper amount of NP solution was loaded into the polymer solutions to get the desired NP concentrations in PNCs. The composite solutions were stirred for another 12 h, and then bath sonicated for 15 min before being cast on glass Petri dishes. The bulk of the solvents were first evaporated in a fume hood at room temperature overnight, and then the samples were vacuum dried in a vacuum oven at 150 °C (above the glass transition temperature of PMMA, *T*_m_ approximately 120 °C, and the standard boiling point of the solvent, *T*_b_ approximately 82 °C ) for 72 h followed by 3 h of annealing at 180 °C to remove the residual solvent.

### 2.3. Morphological analysis

Scanning electron microscope (SEM) images were taken by a Zeiss Ultra Plus Field Emission Scanning Electron Microscope to assess the quality of nanoparticle dispersion in the composites. Sample films were freeze-fractured using liquid nitrogen and the cross-sections were coated with an approximately 8 nm gold layer before the imaging.

### 2.4. Small-angle X-ray scattering

The small-angle X-ray scattering (SAXS) data on nanocomposites were collected using Anton Paar’s SAXSpoint 5.0 at a sample-to-detector distance of 1 m. The samples were sealed between Kapton films and measurements were performed at 25 °C. The static SAXS profiles were used to evaluate the state of nanoparticle dispersion and dispersion stability in the nanocomposites. Frames were collected for 10 min over a total duration of 30 min per sample. The transmission data was also obtained for each sample and the Kapton background for proper data reduction and subtraction. The data reduction was performed using the SAXS analysis program of SAXSpoint 5.0 [28].

### 2.5. Thermogravimetric analysis

Thermogravimetric analysis (TGA) of the samples was studied by TGA Q500 TA Instruments in the temperature range of 25–600 °C under a nitrogen atmosphere with a scan rate of 5 °C/min [37] to verify the mass content of silica NPs and nonlinear BB polymer chains in the composite.

### 2.6. Differential scanning calorimetry

The glass transition temperature (*T*_g_) of the neat polymer and PNCs were determined using differential scanning calorimetry (DSC) [29]. DSC experiments of the pure PMMA, PMMA/silica composites were carried out with DSC Q2000 TA Instruments using approximately 8–10 mg of materials in aluminum pans provided by TA Instruments. An empty aluminum pan was used as a reference. To eliminate the temperature history, the samples were first heated to 180 °C and allowed to remain there for 5 min isothermally. Samples were then cooled down to 25 °C at the cooling rate of 20 °C/min. The heat flow curves were then collected while heating the samples at the ramp rate of 10 °C/min up to 180 °C. *T*_g_ is estimated from the inflection point at the transition.

### 2.7. Rheology

Linear viscoelastic rheological measurements in the melt state are performed at 150, 175, and 200 °C, and the standard time-temperature-superposition (TTS) principle was applied to obtain the master curves with a reference temperature of 200 °C [38]. The measurements were performed on an Anton Paar MCR 302 rheometer equipped with a parallel plate geometry with a diameter of 8 mm. For small-amplitude oscillatory shear (SAOS), amplitude sweep tests were first performed from 0.01% to 10% strain at ω = 100 rad/s deformation frequency to determine the linear viscoelastic region (LVR) for each temperature. The frequency sweep measurements were performed at stain amplitudes in the LVR and at frequency ranges from 0.01 to 100 rad/s at LVR for 200 °C and from 0.1 to 100 rad/s for other temperatures.

The TTS principle relates the frequency ω dependence of the complex modulus, i.e. G*(ω), at any temperature to a reference temperature (which is taken to be 200 °C in this study) where a frequency-scale shift factor (a_T_) and a modulus-scale shift factor (b_T_) can be used to superimpose the frequency-dependent data at temperature T with the data at the reference temperature.


(1) $$G^{*}(\omega ;T)=b_{T}\;G^{*}(\omega a_{T};T_{ref})$$

The temperature dependence of the a_T_ is empirically described by the Williams-Landel-Ferry (WLF) equation [39]:


(2) $$\text{log}(a_{T})=\frac{-C_{1}\;(T-T_{ref})}{C_{2}+(T-T_{ref})},$$

where T is the experiment temperature, T_ref_ (200 °C in this study) is the reference temperature chosen to construct the compliance master curve and C1, and C2 are empirical constants adjusted to fit the values of the superposition parameter a_T_ to the WLF equation. C1 and C2 constants for each fit are given in Table S1 in supporting information (SI). Based on the calculated constants and T_g_ of the samples the fragility index (m) [40, 41], of the chains can be estimated from the equation below:


(3) $$m=\frac{C_{1}\;T_{g}}{C_{2}}$$

### 2.8. Broadband dielectric spectroscopy (BDS)

Broadband dielectric relaxation spectra were obtained in the frequency range of 10^−2^ − 10^−7^ Hz using an Alpha A analyzer (Novocontrol Co.) equipped with ZGS Active Cell and Quatro temperature control unit (Concept 40) [42]. BDS measurements for linear and nonlinear neat PMMA and PMMA/silica composites were performed at 50, 100, 150, 175, and 200 °C, respectively. At each temperature increase, the experiments were started once the temperature stability was reached within 0.05 K interval. The data was collected using WinDETA software and the fittings were performed using WinFIT software.

## 3. Results

**Figure 1:**
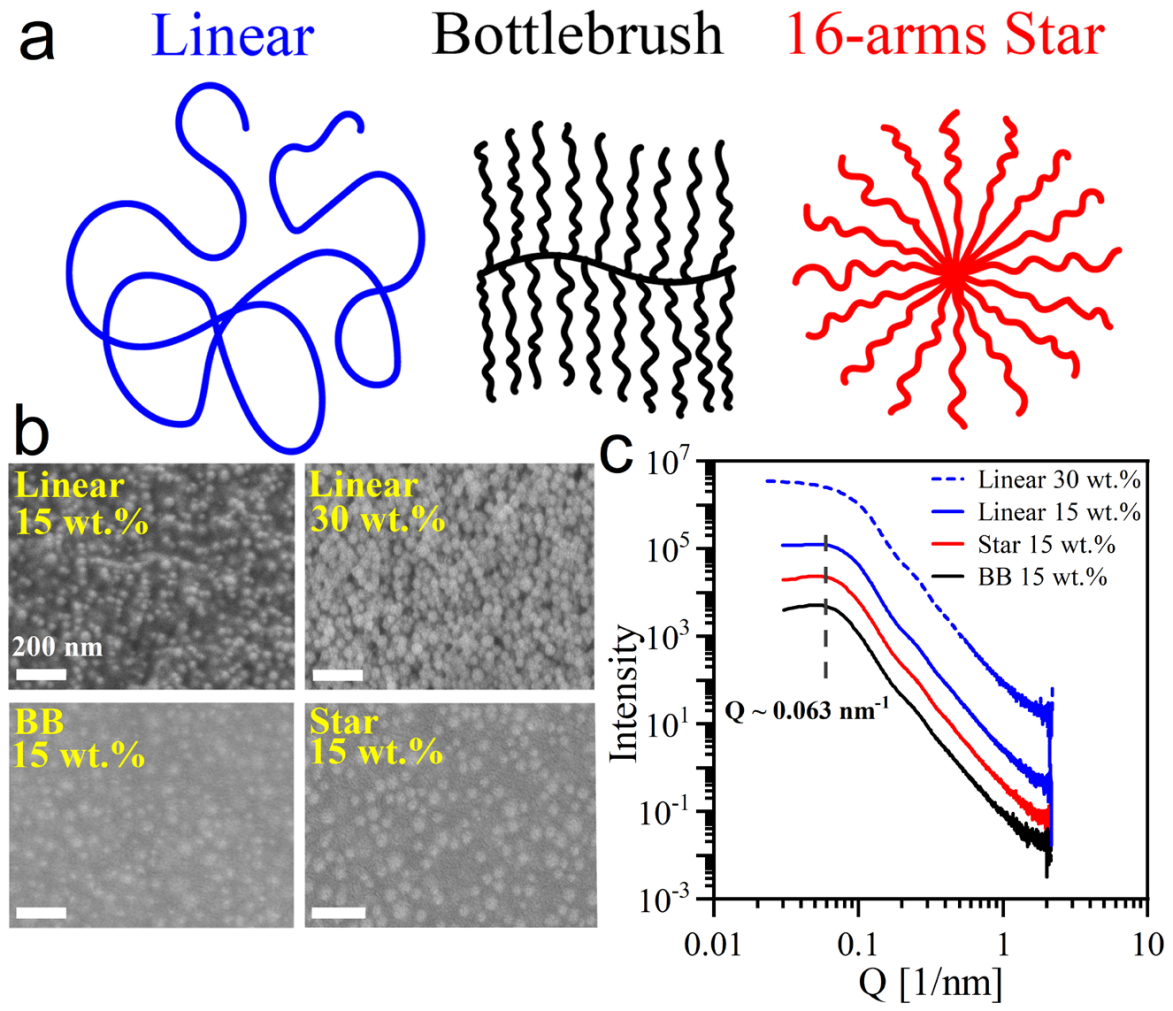
a) Schematic representation of linear (blue), bottlebrush (black), and 16-arms star (red) architectures. b) SEM images of PMMA/silica composites. White bars show a 200-nm scale. c) The SAXS intensity profiles of the nanocomposites obtained for 15 wt.% SiO_2_ loading shows individual particles’ dispersion in the PMMA matrices with different architectures. However, profiles of the aggregated composite (linear PMMA with 30 wt.% silica) show an upturn in the low Q domain with no distinct peak. The profiles are shifted vertically for clarity.

Fifty nanometers diameter silica NPs and poly (methyl methacrylate) with linear, 16-arms star, and bottlebrush architectures (with the same total molecular weight of 120 kDa) were used to prepare the PNCs and compared the properties of PNCs with respective neat (particle-free) polymers. A schematic representation of PMMA architectures is shown in Figure 1a, and their characteristics are given in Table. PMMA-silica is a model attractive system where carbonyl oxygen on the backbone of PMMA can form hydrogen bonding with NPs through surface hydroxyl. This attractive interaction between PMMA chains and silica nanoparticles leads to the formation of an adsorbed polymeric layer on the surface of NPs and allows sterically protected NPs to disperse well in the linear PMMA matrix [43]. Here, the dispersion of NPs in different PMMA architectures and the interface effects on bulk rheology and segmental dynamics are investigated in the first part of the results section. Sample preparation techniques play a critical role in the development of PNCs with desirable properties [11, 12]. One of the key challenges in preparing NP composites is achieving uniform dispersion of the nanoparticles within the polymer matrix. When the NP dispersion is poor, the resulting composite material may exhibit reduced mechanical strength, poor electrical conductivity, or other undesirable properties. To address this challenge, a variety of sample preparation techniques have been developed to achieve a high degree of NP dispersion within polymer matrices. Melt-mixing, solution-mixing, in situ polymerization, freeze drying, electrospinning, and selective laser sintering techniques are the most commonly used techniques to produce polymer nanocomposites with a variety of NP materials, including silica particles, metal nanoparticles, and carbon nanotubes [12]. For the PMMA-silica system, the solvent casting method is known to provide good NP dispersion [14], this method is being used in this study to prepare the samples.

Sample preparation techniques are crucial for developing polymer nanocomposites (PNCs) with desired properties. Achieving uniform dispersion of nanoparticles (NPs) within the polymer matrix is a significant challenge. Poor NP dispersion can lead to reduced mechanical strength, inadequate electrical conductivity, and other unfavorable properties in the resulting composite material. To overcome this challenge, various sample preparation techniques have been developed to achieve a high degree of NP dispersion in polymer matrices. Commonly used techniques include melt-mixing, solution-mixing, in situ polymerization, freeze-drying, electrospinning, and selective laser sintering. These techniques can be applied to different NP materials such as silica particles, metal nanoparticles, and carbon nanotubes. For the specific PMMA-silica system, the solvent casting method, known for providing good NP dispersion, is being employed in the current study for sample preparation.

### 3.1. Dispersion of nanoparticles in PNCs

SEM micrographs of the composites with linear and branched polymers are presented in Figure 1b. For linear polymer, two composites with 15 and 30 wt.% silica were prepared, and NP aggregates were observed at the higher NP concentration, while the composite with 15 wt.% silica loading showed single NP dispersion. As the bulk rheological properties and the chain dynamics are strongly influenced by the dispersion state of the nanoparticles, and the aim is to investigate the role of polymer architecture rather than the NP structuring, it is critical to have similar dispersion states in PNCs. Therefore, the PNCs at 15 wt.% loadings were investigated for the dynamical part.

SAXS intensity profiles of the bulk PNCs (Figure 1c) were also obtained to further verify the dispersion states as SAXS uniquely and noninvasively provides the average interparticle spacings and signatures of clustering if there are any. The lack of intensity upturn in the low Q region confirms that there are no structures larger than the single NP size in 15 wt.% samples. The structure factor peaks in their SAXS intensity profiles of these composites are located at *Q* approximately equal to 0.063 nm^−1^ corresponding to an average spacing of 2π /*Q* ≈ 99 nm between randomly dispersed nanoparticles with 50 nm diameter. This spacing is consistent with the theoretical prediction for the center-to-center interparticle distance ( *L* = *d*[2/(*πϕ*)]^1/3^ ≈ 96 nm for 15 wt.% composites of spherical nanoparticles with the same diameter and concentrations. For the composite with linear PMMA matrix and 30 wt.% silica concentration, a slight upturn in the low-*Q* region was detected, which is an indication of cluster formation in this sample, although it is not very severe. A combination of SAXS, SEM, and theoretical prediction confirms the random dispersion of NPs in all 15 wt.% composites. Hence, the potential differences in the thermal and mechanical properties of these composites can be directly attributed to the difference in the architecture of the polymer matrix chains.

### 3.2. Thermal analysis of homopolymers and composites

Differential scanning calorimetry (DSC) was used to find the glass transition temperature (*T_g_*) of linear and branched chains in their neat and composite forms. The measured *T_g_* values for the PNCs and their neat polymers are given in Table. The results show that the architecture of the polymers and the presence of NPs slightly alter the *T_g_* of the polymers. The neat PMMA has *T_g_* approximately equal to 115.2 °C, whereas there are about 2 °C and 5 °C increase in *T_g_* for the BB and star polymers, respectively. This large shift observed for star PMMA is due to its high functionality resulting in dense monomer packing at the core region, which is lacking in linear and BB architectures. The small increase of *T_g_* in BB is due to the densely grafted side chains, which restricts mobility along the linear backbone and excess free ends which facilitates relaxation. The fact that the *T_g_* value of BB polymer is comparable to the *T_g_* of linear PMMA suggests that the *T_g_* of the bottlebrush polymer is dictated by the side chains and is not significantly influenced by backbone chemistry and length [44]. In all nanocomposite samples, there is a slight increase in *T_g_* (approximately 1 °C for the star, approximately 2 °C for linear, and approximately 3 °C for BB) due to attractive interaction of the PMMA segments and the NPs, which slowdowns the mobility near the interface. Moreover, thermogravimetric analysis (TGA) was performed to verify the mass content of silica NPs and nonlinear BB polymer chains in the BB/silica composite. Figure S1 in SI shows the TGA thermograms of residual weight as a function of temperature for pure silica NPs along with neat BB polymer and 15 wt.% BB/silica composite. The TGA curves for the neat polymer and composite show complete homogeneity because the degradation process of PMMA chains occurs in a single step. The mass loss for neat PMMA begins at about 210 °C and continues until 400 °C. The nanocomposite sample is even more stable due to the presence of NPs; the degradation starts at about 260 °C—well above the temperature range used for rheology and DSC. There is still approximately 15% silica left in the composite sample at 600 °C, validating the reported NP content (15 wt.%).

### 3.3. Rheology of linear and branched PMMA polymers and their nanocomposites

**Figure 2:**
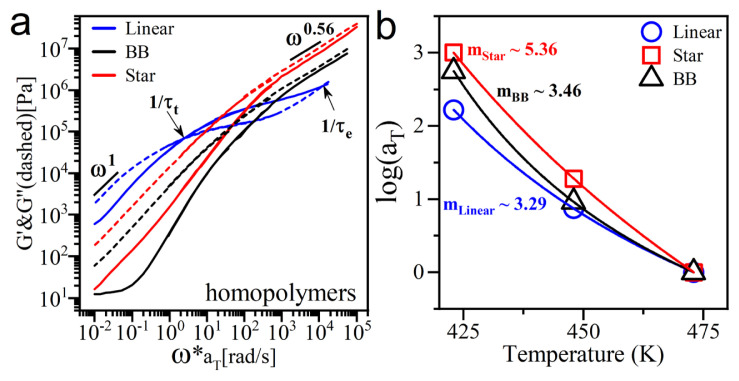
a) Master curves of the neat PMMA polymers with linear (blue), bottlebrush (black), and 16-arms star (red) architectures at a reference temperature of 200 °C. Master curves are compared to see the effect of different architectures on the viscoelastic behavior of the samples. b) Time-temperature superposition shift factors for neat samples along with calculated fragility value. G’ and G” are shown as solid and dashed lines, respectively.

Understanding the role of polymer chain architecture on matrix entanglement and relaxation processes in neat polymers and polymer-nanoparticle composite melts started with identifying the differences in the rheological behavior of PMMA homopolymers with different architectures. Master curves of linear viscoelastic moduli in small amplitude oscillatory shear regimes for neat linear, BB, and 16-arms star PMMA melts are presented in Figure 2a. The time-temperature superposition (TTS) principle is used to determine the temperature-dependent linear viscoelastic behavior of the homopolymers at the reference temperature of 200 °C. The TTS procedure yields continuous dynamic master curves that encompass the entire relaxation profile of the polymers from Rouse dynamics to terminal flow. Figure S2 in SI shows the superposed G’ and G” curves for neat linear PMMA melt at three different temperatures. Both storage and loss moduli of the sample increase when the temperature is decreased. Since the curves of the instantaneous modulus as a function of time do not change shape as the temperature is reduced, the master curve was obtained only by applying shift operation in the frequency domain, and no vertical shift was needed, as expected.

In parallel with the previous rheological analysis of entangled PMMA melts [2, 45, 46], three sequential relaxation regions are identified for neat linear PMMA. Rouse regime, entanglement plateau, and terminal regime. Immediately after the entanglement relaxation time (τ_e_) (crossover between storage and loss modulus at the high frequency), a plateau-like behavior is observed in the intermediate frequencies due to the confined motion of the entangled chains within the entanglement meshes. The behavior is solid-like (G’ exceeds G”) due to the transient network formed. Note that the entanglement molar mass of PMMA is approximately 10 kg/mol [47]. This region is missing in the master curve of unentangled short chains while increasing the molecular weight of the linear polymers extends the entanglement region and delays the whole chain relaxation [16]. At the end of the entanglement plateau, there is another crossover point in the low-frequency domain which identifies the terminal relaxation (τ_e_) and beginning of the terminal regime, governed by the center of mass of the whole polymer chain.

Branched polymers with long side chains (M_arm_ > M_e_) also show three relaxation regions: namely, Rouse regime, arm regime, and terminal regime. An intermediate regime appears after following the Rouse regime and before the terminal flow, which describes the relaxation of the side chains (or arms). The extent of this arm regime depends on the length of the side chains and the backbone to which sidechains are attached [20, 48, 49]. However, the length of the branches in the BB and star polymers described here are slightly shorter than the entanglement length of the corresponding linear polymer, which for PMMA is approximately 10 kg/mol [47, 50]. Therefore, only a weak arm regime is observed for these samples with no sign of entanglement plateau, as reported in previous studies of other BB and star polymers [20, 21, 30, 36, 49]. This part will be discussed in more detail later in the text. The master curve of BB and star polymers demonstrate a predominantly viscous response to the imposed deformation, as G” remains greater than G’ at all frequencies. The observed nearly parallel dynamic moduli in master curves of these branched polymers follow the power law: G’ ~ G”~ω^0.56^, which is very close to that predicted by the Rouse model for unentangled polymer melts (G’~ G”~ ω^0.5^) [16]; therefore, the BB and star polymers are unentangled, and the observed shoulder in the high-frequency domain describes the dynamics of the orientation of the chains in the sample and can be characterized by steady-state recoverable creep compliance [51]. Comparing the branched samples, a higher modulus is measured for the star architecture due to the longer side chain length in this polymer (M_arm_ approximately 7.5 kDa) compared to the BB chains (M_branch_ approximately 5 kDa). Also, the long arm in star chains enhances the inter penetrability among the star chains, which leads to higher G’ and G”.

Further analyzing the rheology data showed that PMMAs with different chain architectures exhibit dynamic responses with distinct temperature dependence. The TTS analysis shows negligible modulus-scale shifts (b_T_ approximately equal to 1) and comparatively large frequency-scale shifts (a_T_) which were fitted to the WLF equation and presented in Figure 2b, along with their calculated fragility index (m). In homopolymers with BB and star structures, a_T_ shows stronger temperature dependence and fragility compared to the linear PMMA, suggesting the larger activation barrier for segmental dynamics and enhanced dynamical heterogeneities.

The same linear, BB, and star polymers were used to prepare PMMA/silica nanocomposites with 15 wt.% silica loading (see the sample preparation section for more details). The temperature-dependent horizontal shift factors and WLF fits for the composites are presented in Figure S3, and their fragility index is listed in Table S1. Adding NPs slightly increases the horizontal shift factor for the composites with branched matrices and slightly decreases the fragility index. The linear viscoelastic behavior of PNCs is governed by matrix and particle microstructure. Since silica nanoparticles are well dispersed in the PMMA matrices, the microstructure contribution is not a dominant factor. Still, the effect of NP concentration in the PNC models with linear PMMA matrices is investigated where clustering was observed in 30 wt.% loadings. Figure S4 compares the master curves of the neat linear PMMA and respective PMMA/silica nanocomposites. Adding NPs and increasing NP concentration reinforces the PNCs that cause an increase in both storage (G’) and loss (G”) moduli. While the 15 wt.% composite with a well-dispersed NP network shows a moderate increase in G’ and G” in the low-frequency domain, the 30 wt.% composite shows dramatically higher moduli compared to the homopolymer, with G’ > G” in all frequency domains, which is an indication of solid-like nature of this system due to the agglomeration of NPs in the matrix [7, 52] (see the SAXS intensity plot for the 30 wt.% samples).

**Figure 3:**
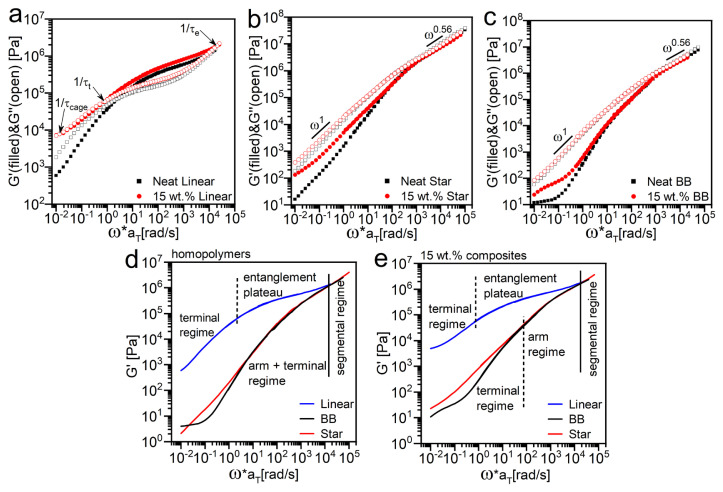
Master curves of the neat PMMA polymers with (a) linear, (b) star, and (c) bottlebrush architectures are compared with the master curves of their 15 wt.% composites at a reference temperature of 200 °C. Storage modulus master curves of (d) neat and (e) composites samples shifted vertically to overlay at the edge of the segmental regime.

Focusing on the well-dispersed samples, Figure 3 compares the master curves of the neat polymers with linear, star, and BB architectures and their corresponding 15 wt.% composites. Similar to the reports from previous studies [2, 53, 54], by dispersing NPs in the linear PMMA matrix, the terminal relaxation time is slightly shifted to lower frequencies, the rubber plateau modulus only slightly increased, the entanglement region extended, and the frequency dependence of the storage modulus is decreased (see Figure 3a). With the addition of nanoparticles, another crossover between storage and loss modulus appears at the lowest frequency, which is defined by (τ_cage_) and represents the relaxation time of the particle network mediated by the interfacial polymer [2, 55]. For the star and BB macromolecules with unentangled side chains, the effect of adding NPs becomes evident in the terminal regime where the storage modulus of composite deviates from the moduli of the homopolymers, as shown in Figures 3b and 3c.

The reinforcement factors (here defined as the ratio of G’ of the composites over the G’ of their neat polymer counterparts) are plotted in Figure S5 to better evaluate the effect of NPs on the rheology of the composites at different deformation frequencies. Although the storage modulus for the composite with linear chains is slightly higher than the G’ of the neat sample, the reinforcement factor is nearly the same for all three composites in the high-frequency domain, suggesting that the average segmental dynamics are not significantly altered by NPs or the small differences are not identified by the bulk rheological measurements. The reinforcement factor starts to increase toward the terminal regime, where the whole chain diffusion is significantly suppressed by the addition of NPs due to the attractive nature of this model composite system which slows down the dynamics of the chains attached to the surface and the polymer-polymer interactions between adsorbed and free chains [4].

The differences between the linear and branched homopolymers and composites are more easily visualized with an overlay of the storage modulus master curves in the Rouse regime (see Figure 3d for linear and Figure 3e for branched polymers). All three polymer melts exhibit the same behavior in the Rouse regime, where the localized relaxations of monomeric segments occur independent of polymer chain architecture. Then, the master curve of linear PMMA shows a clear deviation from bottlebrush and star melts by displaying a rubbery plateau for several decades in reduced frequency (which is characteristic of well-entangled polymer melts), before entering the terminal flow region. On the other hand, the unentangled BB and star polymers exhibit indistinguishable behavior over about five decades due to their branching with short/unentangling side chains. However, a clear deviation between star and BB begins to develop only at the terminal regime in both homopolymer and composite samples. It needs to be noted that although star and BB chains described here have different architectures, the backbone length in BB polymer is quite short, which makes it behave like a star polymer with an elongated core; hence, both branched homopolymers here show very similar rheological characteristics. However, in the composite samples presence of NPs directly affects the whole chain relaxation process, and this feature can help us to separate arm and terminal regimes more accurately. Comparing the storage master curves of branched homopolymers and composites, it is seen that the deviation of the storage modulus between star and BB samples occurs at higher frequencies (short times) for the composites (at ω > 10 rad/s), while the G’ of neat star and BB samples overlap further and it occurs at a lower frequency (ω < 10 rad/s).

### 3.4. Effect of PMMA architecture on the segmental dynamics

**Figure 4:**
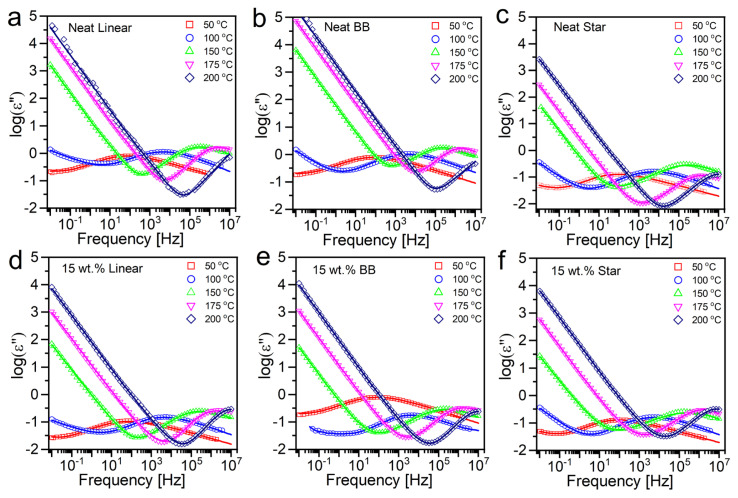
The frequency dependence of the dielectric relaxation data at 50, 100, 150, 175, and 200 °C along with HN fits in linear and nonlinear neat PMMA and PMMA/silica composites.

Since rheology does not give a sufficient resolution to distinguish the effects of polymer architecture on the segmental dynamics, broadband dielectric spectroscopy (BDS) has been employed. The dielectric relaxation of pure PMMA has been studied by several research groups in the last decades [45, 56, 57]. Our aim here is to understand the influence of silica nanoparticles and different polymer topologies on the dynamics. Figure 4 displays representative dielectric loss spectra along with Havriliak-Negami (HN) function fits for the neat linear (Figure 4a), star (Figure 4b), and BB (Figure 4c) PMMAs and their respective 15 wt.% composites (shown in Figure 4d for linear, Figure 4e for star, Figure 4f for BB composites) at five distinctive temperatures, specifically 50, 100 °C (lower than *T*_g, PMMA_) and 150, 175, and 200 °C (higher than *T_g_*_, PMMA_). At temperatures below *T*_g, PMMA_, (50 and 100 °C), the dielectric loss spectra of neat and composites samples show two processes: β-relaxation, and very low DC conductivity. By increasing the temperature, the β-relaxation peak moves to the higher frequencies which is an indication of an increase in the dynamics of the rotations in the chain backbone. The origin of the β process is the partial rotation of the −COOCH_3_ side groups around the C−C bonds which links these side groups to the main chain. By increasing temperature, the side chain movement implies some coordinated motion with neighboring side chains and ultimately merges with the micro-Brownian motions of the main chain backbone (α process) at temperatures above *T*_g_ [29]. Hence, at 150, 175, and 200 °C the α-relaxation of PMMA is also detected for all three architectures in both neat and composite samples. Therefore, at low temperatures (lower than *T*_g, PMMA_), only the β process was detectable and one HN function was sufficient to define the PMMA dynamics, whereas, at high temperatures (higher than *T_g_*_, PMMA_), two HN functions were required to define the relaxation involving β and α processes. In the frequency domain, the HN function is given as:


(4) $${ɛ}^{*}={ɛ}_{\infty }+\frac{\Delta ɛ}{{\left[1+{\left(\frac{i\omega }{\omega_{0}}\right)}^{a}\right]}^{b}},$$

where *ω* = 2*πf* is the angular frequency, and *ω*_0_ = 2*πf*_0_ is a characteristic angular frequency related to the dielectric loss peak frequency (*f_max_*). ). The relaxation strength (Δ*ɛ*) is the difference between the real permittivity for low-frequency limiting value and that for high-frequency limiting value *ɛ*_∞_. The HN fitting parameter ‘a’ defines the broadness of the relaxation spectrum, while the parameter ‘b’ describes its asymmetry, (0 < a, b < 1).

Since the β process only describes the relaxation of side groups in a very short time and length scale, it is independent of polymer chain architecture and will not be the focus of this work. However, the α process describes relaxations in larger scales which are associated with the segmental motion of the polymer chains and can be affected by the polymer chain architecture and polymer-NP interactions. Finally, it was noted that the segmental dynamics of the polymer chains are highly dependent on the architecture of the polymers. The α process relaxation time in star polymer is more than one other of magnitude higher when compared to linear and BB homopolymers. As mentioned earlier, the cocentric distribution of the arms in star architecture provides a compact and impenetrable core region near the center and restricts the mobility of the arms by steric repulsion. Therefore, arm segments are more difficult to reorient and relax in the core region, whereas in random coils of linear chains, there is no intrachain structuring, thus, the chain segment can relax faster. On the other hand, the side chains along the linear backbone in BB chains can feel the steric repulsion from neighbor branches (which leads to higher activation energy), but their short length and the excess number of unbound free ends yield faster relaxation of the arm segments.

**Figure 5:**
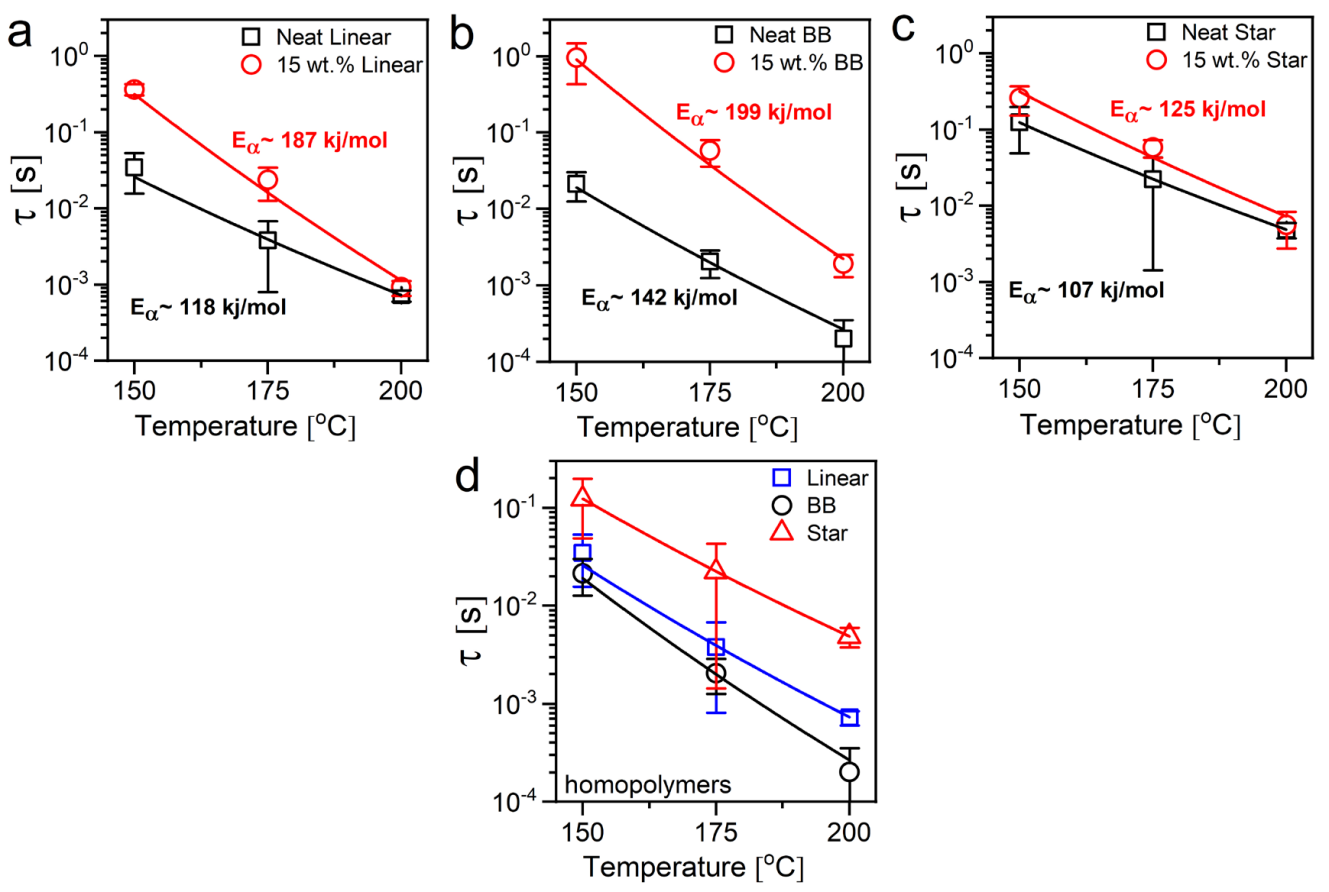
α relaxation time of PMMA chains as a function of temperature for (a) linear, (b) star, and (c) bottlebrush architectures in neat and 15 wt.% composite forms. The figure at the bottom shows the architecture dependence of the α relaxation for homopolymers (d). Lines show the fittings of the Arrhenius equation.

The effect of adding silica NPs into matrices of varying PMMA architecture on the segmental dynamics of the PMMAs is shown in Figure 5. The α relaxation times of linear, BB, and star PMMA chains in their neat and composite forms are compared in Figures 5a, 5b, and 5c, respectively. Multiple weak Van der Waals interactions (e.g., H-bonding) between PMMA segments and silica surface slow down the polymer chains’ segmental mobility, leading to suppressed segmental motion in the composites. For all three architectures, the effect of NPs, the relaxation time for linear and star chains are nearly the same in their neat and composite forms at 200 °C; the reduced mobility of the PMMA segments is more pronounced at lower temperatures. The highest reduction in segmental relaxation by adding silica NPs is observed for the BB PMMA. On the other hand, star PMMA shows the lowest decrease in segmental mobility compared to BB and linear architectures. This is attributed to the difference in the conformation of the chains at the polymer-NP interface due to the difference in the special distribution of the monomers in the star and BB topologies. Cocentral branching in the symmetric star architecture creates an impenetrable and compact core region in the chains, limiting the direct touch between the star polymer’s segments and the silica NPs. On the other hand, linear branching along the linear backbone in BB architectures makes it easier to provide a higher number of contact points with silica surface; hence, it leads to more robust attractive interaction with the NPs, which increases segmental relaxation time.

The BDS data was further analyzed, and it found for all samples above the glass transition temperature that the α-relaxation process follows the Arrhenius behavior expressed as:


(5) $$\tau_{f}=\tau_{0}\;{exp}^{\left(\frac{E_{f}}{RT}\right)},$$

where τ_0_ is the reference time, *E*_¦_ is the active energy of relaxation, and *R* is the gas constant number (8.314 J/mol K). Then, the activation energies (*E*_¦_) for the α-relaxation processes from the Arrhenius fits are also displayed for both neat and composite samples (see Table S2 also). Compared to the neat polymers shown in Figure 5d, higher *E*_¦_ values were observed for the composite samples for all PMMA architectures. The *E*_¦_ increases by approximately equal to 58% in the PNC with linear chains compared to linear homopolymer whereas the branched architectures are less influenced by the NPs. The increase in the activation energy of the composites is primarily due to the attractive interactions between polymer chains and the NPs, which restricts the mobility of polymer segments and demands higher energy to relax. Since the primary mode of interaction between PMMA and silica is through the hydrogen bonding between carbonyl groups of PMMA and hydroxyls in silica, the monomers of the flexible linear chains can interact more easily with the particle surface, thus, their segmental mobility is more affected by the attractive NPs. Overall, these results from multiscale dynamic measurements of the nanocomposites of thermoplastic PMMA polymers with different architectures imply that the methyl rotations of the PMMA backbone (β relaxation) are not significantly altered in the presence of NPs whereas the larger scale dynamics in the molten state, from segmental and reptation to terminal flow, are significantly altered both by NPs. Therefore, changing the polymer chain architecture from conventional linear form to nonlinear architectures can be used as a new design parameter to control the rheological properties of the thermoplastic polymer nanocomposite melts. The mechanical behavior of these nanocomposites in the glassy state is also of considerable interest and will be discussed in future work.

## 4. Conclusion

In this research, we investigated the influence of polymer architecture on the melt rheology and segmental motion of chains in neat form and nanocomposites with spherical silica nanoparticles. Specifically, we employed linear, bottlebrush, and star-shaped PMMA with the same total molecular weight to contribute to the fundamental understanding of how polymer architecture affects the mechanical properties of thermoplastic polymer nanocomposite melts; a property which is lacking in the literature. Our results show that altering the polymer chain architecture from a conventional linear form to nonlinear architecture is an effective approach to controlling the overall mechanical characteristics of thermoplastic polymer nanocomposite melts. SAXS and SEM analysis confirmed random dispersion of silica nanoparticles in all three matrices at 15 wt.% loadings whereas mild aggregation is observed at elevated concentrations. The time-temperature-superposition principle is used to obtain the master curves of dynamic moduli for both homopolymers and composites which covers a wide range of hierarchical relaxation modes, from segmental dynamics to terminal flow. Our rheology experiments demonstrate that branching the polymers without altering the molar mass effectively suppresses interchain entanglement and significantly modifies the linear viscoelastic response of both neat polymers and their composites. Furthermore, our findings suggest that the spatial distribution of branches plays a significant role in determining the mobility of polymer segments. Specifically, we found that PMMA with a symmetric star architecture exhibited the slowest segmental mobility, with an α-relaxation time measured by broadband dielectric spectroscopy that exceeded the times recorded for linear and bottlebrush chains by more than one order of magnitude. Finally, our results indicate that polymer architecture also influences the conformation of chains at the polymer-particle interface and the level of contact between polymer chains and NP surface, which ultimately affects the reduction in segmental dynamics of polymer chains in the presence of attractive nanoparticles. Overall, the outcomes of this study have practical applications in fields such as gas separation and polymeric electrolytes, where the ability to control mechanical properties and multiscale chain dynamics is crucial for designing high-performance composite materials.

## Supplementary Information

**Table S1:** C1 and C2 constants from WLF equation fit along with glass transition temperature and fragility indexes.

Samples	C1	C2 (°C)	T _g_(°C)	m = C1×T _g_/C2
Neat linear	4.02	140.62	115.19	3.29
15 wt.% linear	2.42	98.08	117.57	2.90
Neat BB	3.18	107.83	117.23	3.46
15 wt.% BB	2.52	104.05	120.16	2.91
Neat star	8.73	195.45	120	5.36
15 wt.% star	4.80	135.15	120	4.26

**Table S2:** τ_0_ and values from Arrhenius fits.

Sample	(s)	(kj/mol)
Neat linear	5.7 **×** 10 ^−17^	118.6
15 wt.% linear	2.3 **×** 10 ^−24^	187.3
Neat BB	5.2 **×** 10 ^−20^	142.2
15 wt.% BB	1.9 **×** 10 ^−25^	199.81
Neat star	6.3 **×** 10 ^−15^	107.7
15 wt.% star	1.1 **×** 10 ^−16^	125.4

Figure S1 TGA thermograms of pure silica NP along with neat BB and 15 wt.% BB/silica composite.

Figure S2 Master curve obtained from TTS principle for neat linear PMMA with reference temperature of 200 °C.

Figure S3 Time-temperature superposition shift factors for neat and composite samples with (a) linear, (b) bottlebrush, and (c) star shaped polymers. The lines show the WLF fit to the data at reference temperature of 200 °C.

Figure S4 Master curve obtained from TTS principle for neat linear PMMA and linear PMMA/silica composites with 15 and 30 wt.% silica loadings. The reference temperature is 200 °C for all samples.

Figure S5 Reinforcement in 15 wt.% PMMA/silica composites with (a) linear, (b) star, and (c) bottlebrush chains. Master curve obtained from TTS principle at a reference temperature of 200 °C for all samples.

## Figures and Tables

**Table:** Characteristics of the PNCs and polymers.

Polymers	Sample ID	M_w_ (kDa)	M_branch_ (kDa)	Tg (°C) neat PMMA	Tg (°C) 15 wt.% PNCs
16-arms star	Star	120	7.5	120.2	121.4
Bottlebrush	BB	120	5	117.2	120.2
Linear	Linear	120	60	115.2	117.6
