# Supplementary figures and images for: Polymer architecture effect on rheology and segmental dynamics in poly (methyl methacrylate)-silica nanocomposite melts
Source: Turk J Chem. 2023 Jun 23;47(4):749–62. doi: 10.55730/1300-0527.3576 (PMC10760588; doi:10.55730/1300-0527.3576)

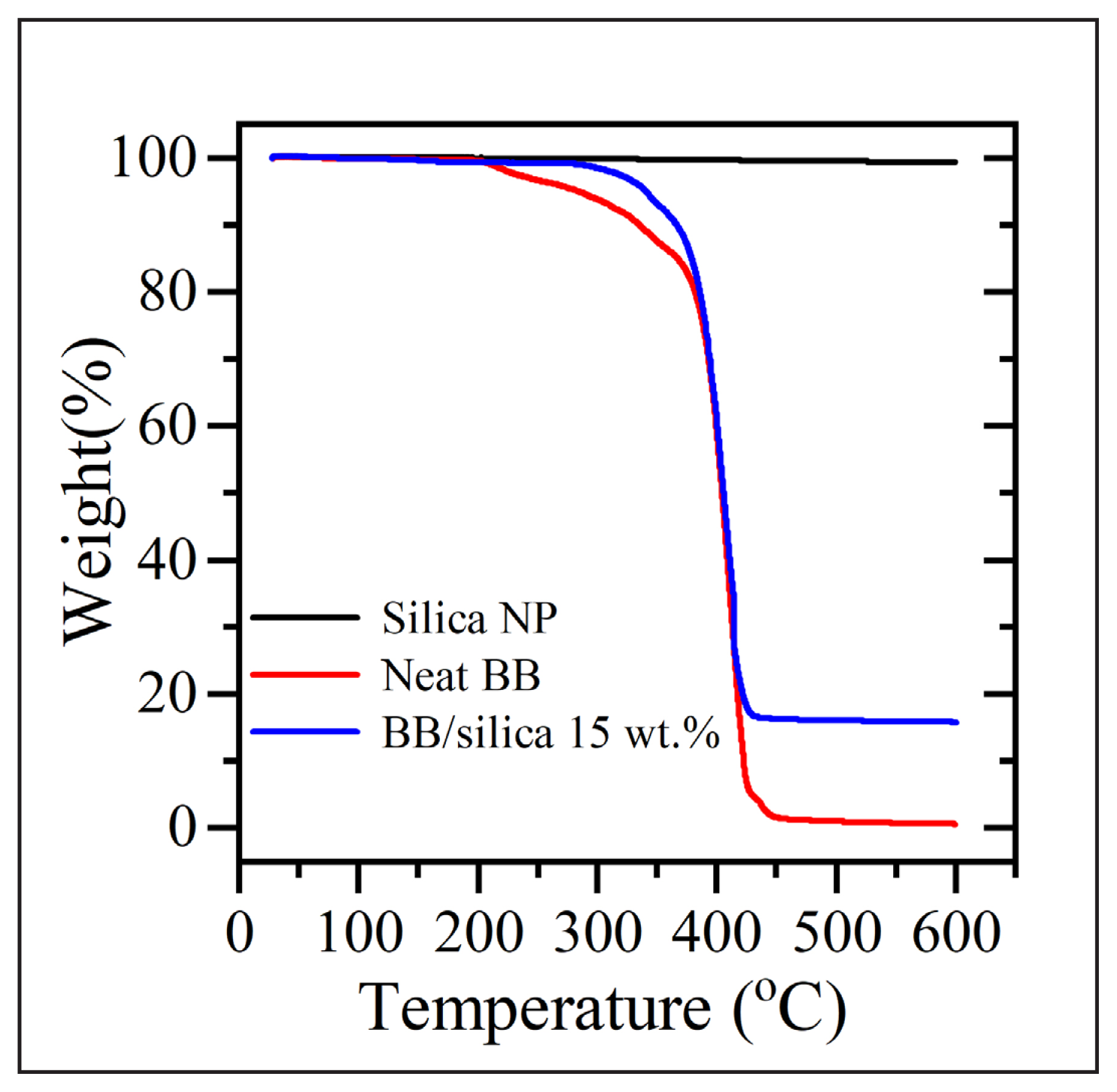

Supplement: Figure S1 — TGA thermograms of pure silica NP along with neat BB and 15 wt.% BB/silica composite. [file turkjchem-47-4-749s1.tif]

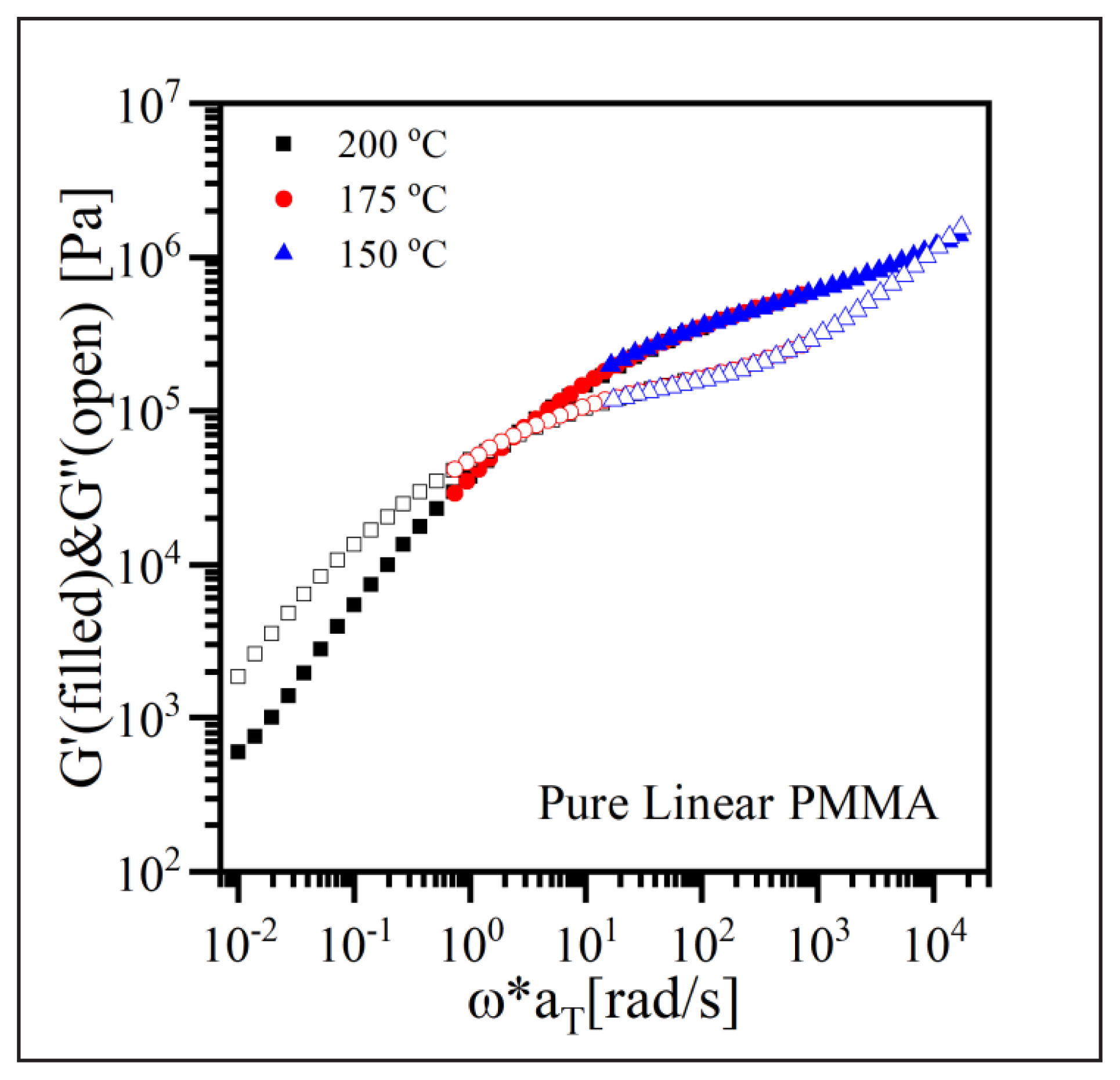

Supplement: Figure S2 — Master curve obtained from TTS principle for neat linear PMMA with reference temperature of 200 °C. [file turkjchem-47-4-749s2.tif]

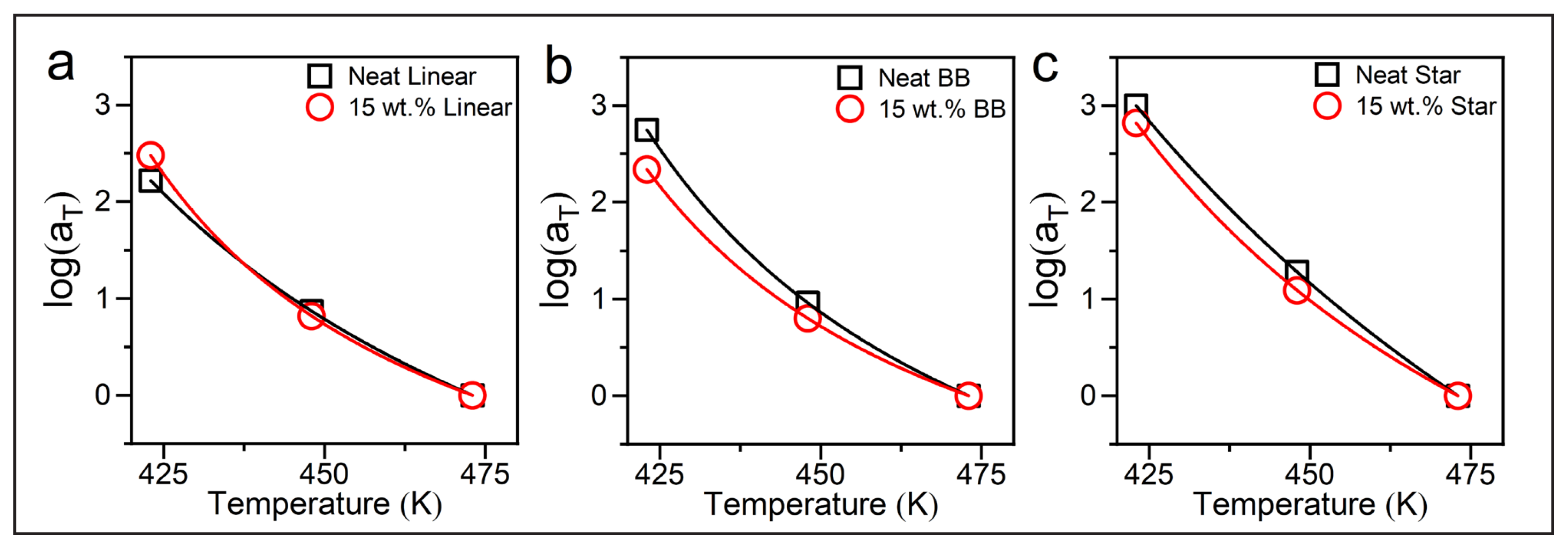

Supplement: Figure S3 — Time-temperature superposition shift factors for neat and composite samples with (a) linear, (b) bottlebrush, and (c) star shaped polymers. The lines show the WLF fit to the data at reference temperature of 200 °C. [file turkjchem-47-4-749s3.tif]

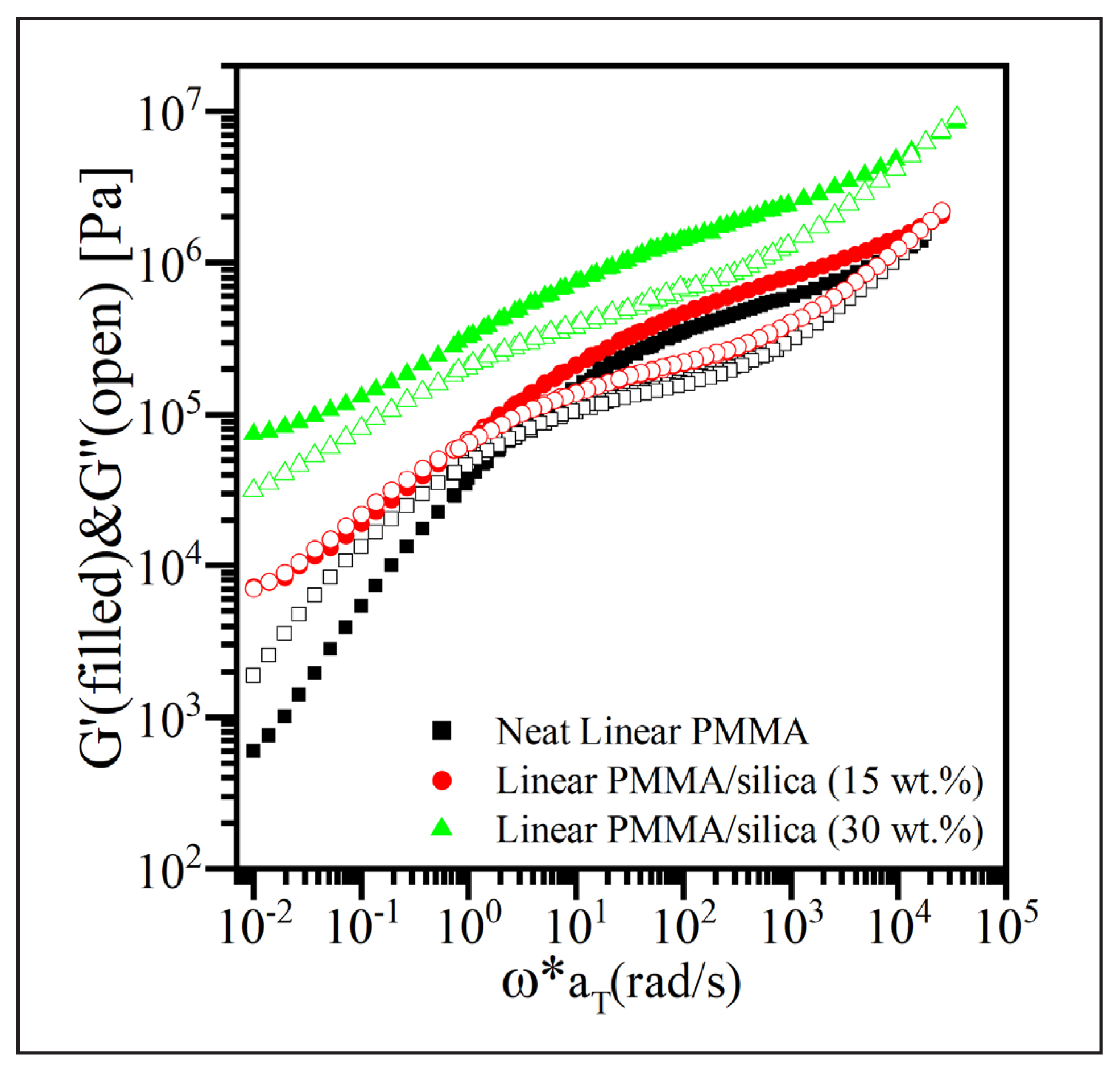

Supplement: Figure S4 — Master curve obtained from TTS principle for neat linear PMMA and linear PMMA/silica composites with 15 and 30 wt.% silica loadings. The reference temperature is 200 °C for all samples. [file turkjchem-47-4-749s4.tif]

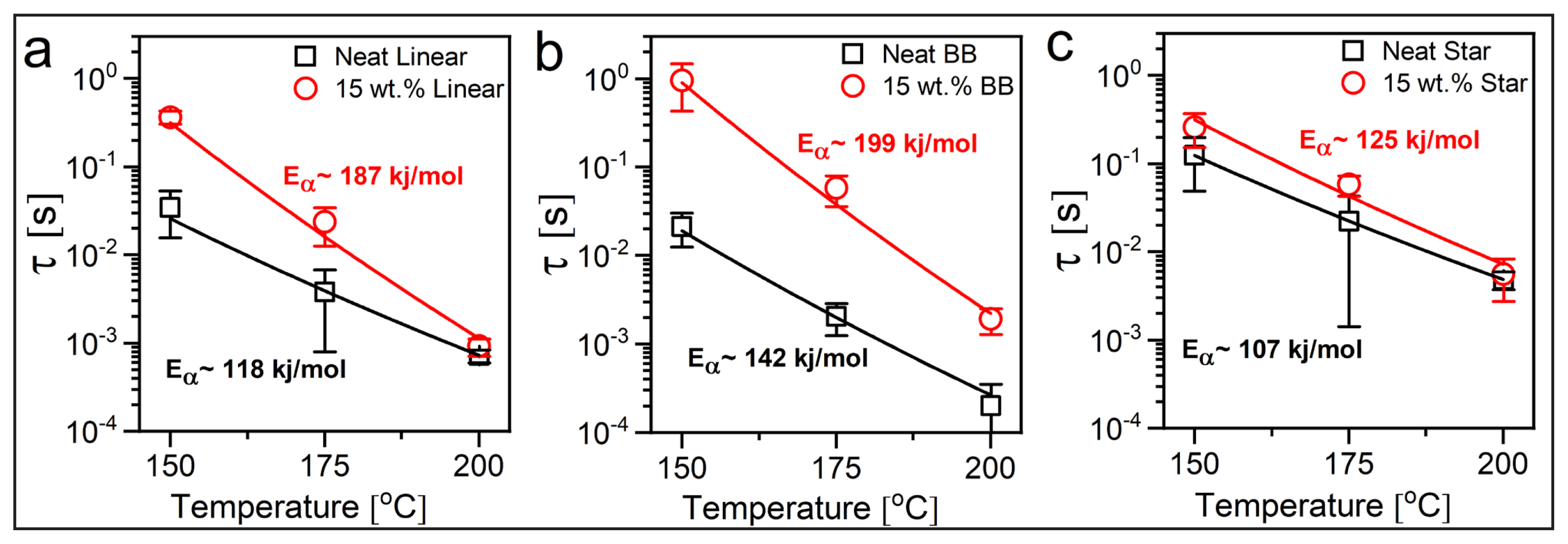

Supplement: Figure S5 — Reinforcement in 15 wt.% PMMA/silica composites with (a) linear, (b) star, and (c) bottlebrush chains. Master curve obtained from TTS principle at a reference temperature of 200 °C for all samples. [file turkjchem-47-4-749s5.tif]
